# Predicting genome-scale *Arabidopsis-Pseudomonas syringae *interactome using domain and interolog-based approaches

**DOI:** 10.1186/1471-2105-15-S11-S13

**Published:** 2014-10-21

**Authors:** Sitanshu S Sahu, Tyler Weirick, Rakesh Kaundal

**Affiliations:** 1 National Institute for Microbial Forensics & Food and Agricultural Biosecurity (NIMFFAB), Oklahoma State University, Stillwater, 74078, USA; 2 Department of Biochemistry & Molecular Biology, Oklahoma State University, Stillwater, 74078, USA; 3 Bioinformatics Facility, Department of Botany & Plant Sciences, Institute for Integrative Genome Biology (IIGB), University of California, Riverside, California, 92521, USA

**Keywords:** Plant-pathogen interactions, Bioinformatics, Unsupervised learning, *Arabidopsis*, *Pseudomonas syringae*, Interactome, Computational prediction

## Abstract

**Background:**

Every year pathogenic organisms cause billions of dollars' worth damage to crops and livestock. In agriculture, study of plant-microbe interactions is demanding a special attention to develop management strategies for the destructive pathogen induced diseases that cause huge crop losses every year worldwide. *Pseudomonas syringae *is a major bacterial leaf pathogen that causes diseases in a wide range of plant species. Among its various strains, pathovar *tomato *strain DC3000 (*PstDC3000*) is asserted to infect the plant host *Arabidopsis thaliana *and thus, has been accepted as a model system for experimental characterization of the molecular dynamics of plant-pathogen interactions. Protein-protein interactions (PPIs) play a critical role in initiating pathogenesis and maintaining infection. Understanding the PPI network between a host and pathogen is a critical step for studying the molecular basis of pathogenesis. The experimental study of PPIs at a large scale is very scarce and also the high throughput experimental results show high false positive rate. Hence, there is a need for developing efficient computational models to predict the interaction between host and pathogen in a genome scale, and find novel candidate effectors and/or their targets.

**Results:**

In this study, we used two computational approaches, the interolog and the domain-based to predict the interactions between *Arabidopsis* and *PstDC3000 *in genome scale. The interolog method relies on protein sequence similarity to conduct the PPI prediction. A* Pseudomonas *protein and an *Arabidopsis* protein are predicted to interact with each other if an experimentally verified interaction exists between their respective homologous proteins in another organism. The domain-based method uses domain interaction information, which is derived from known protein 3D structures, to infer the potential PPIs. If a* Pseudomonas *and an* Arabidopsis *protein contain an interacting domain pair, one can expect the two proteins to interact with each other. The interolog-based method predicts ~0.79M PPIs involving around 7700* Arabidopsis *and 1068* Pseudomonas *proteins in the full genome. The domain-based method predicts 85650 PPIs comprising 11432* Arabidopsis *and 887* Pseudomonas *proteins. Further, around 11000 PPIs have been identified as interacting from both the methods as a consensus.

**Conclusion:**

The present work predicts the protein-protein interaction network between *Arabidopsis thaliana *and *Pseudomonas syringae *pv. *tomato *DC3000 in a genome wide scale with a high confidence. Although the predicted PPIs may contain some false positives, the computational methods provide reasonable amount of interactions which can be further validated by high throughput experiments. This can be a useful resource to the plant community to characterize the host-pathogen interaction in* Arabidopsis *and* Pseudomonas *system. Further, these prediction models can be applied to the agriculturally relevant crops.

## Background

*Pseudomonas syringae *is a Gram-negative bacterium causing economically important diseases in a wide range of plant species leading to severe agricultural losses worldwide. Each strain of *Pseudomonas *shows a high degree of host specificity and infects only a limited number of plant species or even a few cultivars of a single plant species [1,2]. Among them, pathovar *tomato *strain DC3000 (*Pst DC3000*) has been asserted to infect the plant host *Arabidopsis thaliana *and tomato causing bacterial spec and brown spot. Thus, Arabidopsis-Pseudomonas has been accepted as a model system for experimental characterization of the molecular dynamics of plant-pathogen interactions in both resistance and susceptible interactions [1,3,4]. The whole genome sequence of *Pst *DC3000 revealed that it has ~300 virulence-related genes [5]. One of the major classes of virulence factors includes effector proteins that are delivered into the host through a type III protein secretion system (TTSS) to suppress plant immune responses, and also to facilitate disease development [6]. Basically, *Pseudomonas syringae *pathogenesis is dependent on effector proteins and to date, nearly 60 different type III effector proteins encoded by *hop *genes have been identified [http://www.pseudomonas-syringae.org/]. In addition, *Pst DC3000 *also produces non-proteinaceous virulence effectors, including coronatine (COR), which are crucial for pathogenesis. However, the virulence function of a large number of potential effectors encoded by the *Pst DC3000 *genome and their mode of action is still unknown. Similarly, in* Arabidopsis *it has been seen that approximately 3000 proteins are directly related to plant defense [7]. Many of these proteins interact directly with the pathogen proteins and some of them initiate plant defense responses to the infection. Recently, Mukhtar *et al*. [8] reported an experimental protein interaction network (PPIN-1) containing 843* Arabidopsis *proteins and 83 pathogen effectors including very few interactions with *Pst DC3000*. Till now, only nearly 10 % of the full genome of* Arabidopsis *has been evidenced for interaction. Therefore, to functionally characterize the dynamic interactions of plants with bacterial pathogens, there is a need for genome-wide study of the host-pathogen interactions. Knowledge of such novel resistance interactions provides the backbone of the understanding of plant resistance mechanisms and will aid in the further analysis of plant immunity [9].

Generally, pathogen attacks host tissues, secreting degradation enzymes and toxin release. Many of such mechanisms involve the protein-protein interactions (PPIs). PPIs are essential process in all living cells and play a crucial role in the infection process, and initiating a defense response. In this context, understanding the PPI network (interactome) between plant proteins and pathogen proteins is a critical step for studying the molecular basis of pathogenesis [10,11]. In particular, computational approaches ameliorate the study of host-pathogen protein interactions in a genome-wide range.

In the past decade, a series of PPI prediction methods have been elegantly developed and are playing an increasingly important role in complementing experimental approaches. Diverse data types or properties, such as gene ontology (GO) annotations [12], protein sequence similarity [13], protein domain interactions [14], and protein structural information [15,16] have been frequently utilized to construct PPI prediction methods. Among these computational methods, the interolog and the domain-based methods [17-23] are widely used approaches for PPIs prediction.

In this work, we used the interolog and the domain-based methods to jointly predict the protein-protein interactions between *Pseudomonas syringae *and *Arabidopsis thaliana*. The domain-based approach infers inter-species protein-protein interactions by known domain-domain interactions from various databases and the interolog approach identifies protein-protein interactions based on homologous pairs of protein interactions across different organisms. We present the prediction pipeline in detail and the functional analysis of the predicted results.

## Materials and methods

### Data sources

The whole proteome of *Pseudomonas syringae pv. tomato DC3000 *is downloaded from* Pseudomonas *genome database (http://www.pseudomonas.com/download.jsp) which contains 5619 protein sequences. Similarly, the full genome of *Arabidopsis thaliana *containing 35386 protein sequences is extracted from the TAIR10 database (http://www.arabidopsis.org/). To infer the prediction from the interolog, we have used two types of datasets: the HPIDB dataset and DIP dataset. Database of Interacting Proteins (DIP) is a collection of experimental determined interactions between proteins in intra-species [24]. As of Jan 2014, DIP database contains 25749 sequences of 72380 protein-protein interactions. Host Pathogen Interaction Database (HPIDB) is a database of experimental determined interactions between 62 host and 529 pathogens [25]. As of Jan 2014, HPIDB database contains 29922 sequences of 23735 unique protein-protein interactions. To implement the domain based model, the domain-domain interaction databases, iPfam and 3DID are used. The iPfam database is a catalog of protein family interactions, including domain and ligand interactions, calculated from known structures in protein data bank (PDB). As of Jan 2014, the iPfam1.0 database contains 5442 domain-domain interactions. The database of three-dimensional interacting domains (3DID) is a collection of high-resolution three-dimensional structural templates for domain-domain interactions. It contains templates for interactions between two globular domains as well as novel domain-peptide interactions. As of Jan. 2014, the 3DID database contains 8323 domain-domain interactions.

### Identification of secreted proteins in *Pseudomonas syringae*

All proteins of* Pseudomonas *are processed through the Psortb3.0 (widely used tool for protein localization in bacteria [26]) and those predicted as cytoplasmic or cytoplasmic membrane are discarded as these proteins have less chance of involvement in interaction. The rest proteins annotated with extracellular, outer membrane and unknown are considered to be positive candidates for interaction. Again we search the whole proteome of* Pseudomonas *through the effector database (http://www.effectors.org/) [27], which is an integrated database for secreted type proteins for bacteria. Those identified as secreted are considered as positive candidates for interaction. Combining these two steps, 2744 potential candidate proteins of *PstDC3000 *are filtered for interaction prediction.

### Prediction of PPIs between* Arabidopsis *and *Pseudomonas*

**Figure 1:**
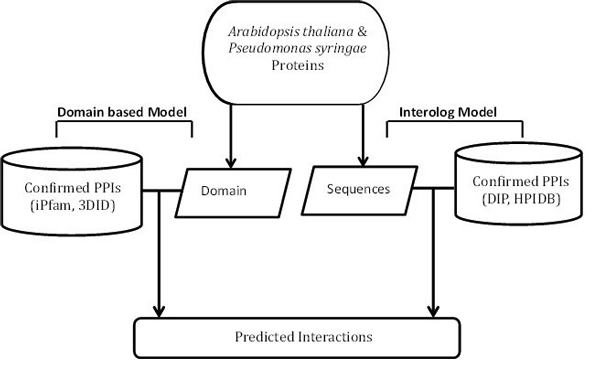
**Overall prediction framework of the interactions between *Arabidopsis thaliana *and *Pseudomonas syringae***.

In this study, the probability of interaction between an* Arabidopsis *and a* Pseudomonas *protein is inferred from two approaches: the domain based and the interolog method individually. The prediction framework is shown in Figure 1.

### Domain based protein-protein interaction prediction

The domain-based method uses domain interaction information, which is derived from known protein 3D structures, to infer the potential PPIs. If two proteins contain an interacting domain pair, it is expected that these two proteins may interact with each other. To get the domains in* Arabidopsis *and Pseudomonas, HMMPfam is used in interproscan5 [28]. In total, 49073 domains are extracted for all the* Arabidopsis *proteins and 7253 domains are collected for *PstDC3000*. If a protein pair between* Pseudomonas *and* Arabidopsis *contains an interacting domain pair from iPfam and 3DID, then the pair is expected to interact with each other.

### Interolog based protein-protein interaction prediction

**Figure 2:**
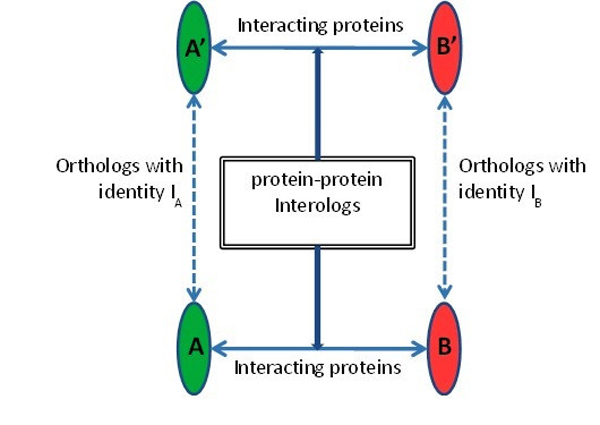
**Illustration of protein-protein interologs**. A and B are two different interacting proteins in one organism and A^' ^and B^' ^are two interacting proteins in another organism. Protein A-A^' ^and B-B^' ^are orthologs between the two organisms. Thus, protein pair A^'^-B^' ^and A-B are interologs and conserved in the organisms.

The interolog method relies on protein sequence similarity to conduct the PPI prediction. An interolog is a conserved interaction between a pair of proteins which have interacting homologs in another organism [29]. The illustration of interolog is shown in Figure 2. Consider that *A *and *B *are two different interacting proteins of one organism, and *A' *and *B' *are two different interacting proteins of another organism. Then the interaction between *A *and *B *is an interolog of the interaction between *A' *and *B'*, if *A *is a homolog of *A', B *is a homolog of *B', A *and *B *interact, and *A' *and *B' *interact. Thus, interologs are homologous pairs of protein interactions across different organisms. Each protein in* Arabidopsis *and* Pseudomonas *is BLASTed against all the protein sequences in the DIP and HPIDB database to identify the homologs with E-value, sequence identity and aligned sequence length coverage of 1.0E^-4^, 50 and 80% respectively. Each protein pair between* Pseudomonas *and* Arabidopsis *is predicted to interact if an experimentally verified interaction exists between their respective homologous proteins in DIP or HPIDB databases.

## Results and discussion

### Prediction of interactions

**Figure 3:**
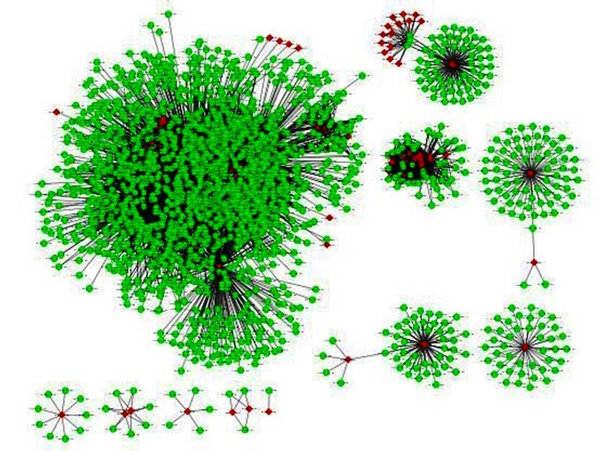
**Visualization of the predicted protein-protein interactions between *Arabidopsis thaliana *and *Pseudomonas syringae***. Each node represents a protein and each edge refers an interaction. Green color circles represent* Arabidopsis *and red color diamonds represent Pseudomonas. The network is generated using the Cytoscape tool.

To predict the genome wide interactions, all proteins of* Arabidopsis *and* Pseudomonas *are paired up, which constitute ~97M PPIs. The interaction probability of each pair is assessed through the domain-based model and interolog-based model separately. The predicted interactions from these methods are reported in Table 1. A total of ~0.86M probable PPIs are predicted from both the methods, which include ~14043* Arabidopsis *proteins and 1337 Pesudomonas proteins. Out of these, 85650 PPIs are predicted by domain based method involving 11432* Arabidopsis *and 887* Pseudomonas *proteins. Similarly, the interolog method predicted ~0.79M PPIs including 7766* Arabidopsis *and 1068* Pseudomonas *proteins. Nearly, 11000 PPIs are consistently predicted by both methods as consensus which comprises 2043* Arabidopsis *and 93* Pseudomonas *proteins. The interaction network of the consensus predicted PPI is shown in Figure 3. On average, a* Pseudomonas *protein has around 118* Arabidopsis *interacting partners, whereas an* Arabidopsis *protein interact with around 6* Pseudomonas *proteins. The reported results are coherent with the previous studies in which it is demonstrated that a few pathogen proteins involved in interaction in the host interactome [11,18,19]. All predicted interactions from the domain based method, interolog method and the consensus predictions are available in Tables S1-S3 respectively in Additional files 1, 2 and 3.

**Table 1:** **Prediction results of* Arabidopsis *and *Pseudomonas syringae *interactions using domain and Interolog approaches**.

Predicted PPIs	# of PPIs	#* Arabidopsis *protein involved	# of* Pseudomonas *proteins involved
Domain based method	85650	11432	887

Inerolog method	794006	7766	1068

From Both Methods	868645	14043	1337

Consensus Interactions	11011	2043	93

### Predicted effector hubs

The effectors of* Pseudomonas *with the highest number of edges (hubs) are PSPTO_0135, PSPTO_0400, PSPTO_0540, PSPTO_0808, PSPTO_1510, PSPTO_2303, PSPTO_2529, PSPTO_2632, PSPTO_3161, PSPTO_3583, PSPTO_3890, PSPTO_3912 and PSPTO_4001 with more than 400 PPIs in the Arabidopsis-Pseudomonas interactome. There are also several effectors with more than 40 predicted PPIs. These are PSPTO_4497, PSPTO_1482, PSPTO_4868, PSPTO_4602, PSPTO_3882, PSPTO_0405, PSPTO_1492, PSPTO_4093, PSPTO_1949, PSPTO_4776, PSPTO_3130, PSPTO_3900, PSPTO_5014 and PSPTO_4090. In contrast to these hub proteins, several effectors are predicted to interact with very few proteins. These hub proteins play important role in pathogenesis, hence can be further investigated for deciphering virulence mechanism.

### Functional enrichment analysis of proteins involved in the Interaction

Functional enrichment analysis is an important assessment for elucidating the functional relevance of the host and pathogen proteins involved in the PPIs. The presence of enriched (over-represented) functional categories that are closely related to host defense and pathogen infection support the validity of the predicted PPIs of the prediction models. Gene ontology (GO) is a comprehensive functional system to annotate the gene products. We used the biological process GO term enrichment to see the relevance of the predicted proteins. The Database for Annotation, Visualization and Integrated Discovery (DAVID) is used to conduct the enrichment analysis[30]. The over represented biological processes of* Arabidopsis *and* Pseudomonas *proteins in the predicted PPIs are listed in Tables 2 and 3 respectively. The enrichment analysis in* Arabidopsis *shows that many proteins involved in the biological process, response to cadmium ion and metal ion. In literature, it has been shown that metal ions are required for pathogen virulence and plant defense [31,32]. Fones et al. demonstrated Zn, Ni or Cd are accumulated when Thlaspi caerule resist to a leaf spot caused by *Pseudomonas syringae *pv. *maculicola *[31]. Block and James reveal that the plant immune responses include deposition of lignin and callose in the cell wall and production of reactive oxygen species and anti-microbial compounds [33]. Qiu et al. [34] show that MAPK/ERK Kinase may directly or indirectly act through another signaling cascade to activate a transcription factor. The transcription factor will then bind a particular region of DNA, resulting in the recruitment of RNA polymerase to transcribe a gene that will ultimately contribute to altering the function of the cell and cause pathogenesis[35]. These evidences in literature support our predicted results.

**Table 2:** **Enriched GO biological process terms involved in predicted* Arabidopsis *proteins**.

GO id	GO term	*p*-value
GO:0006468	protein amino acid phosphorylation	8.24E-96

GO:0001906	cell killing	5.62E-28

GO:0006796	phosphate metabolic process	3.17E-45

GO:0007169	transmembrane receptor protein tyrosine kinase signaling pathway	3.16E-56

GO:0007166	cell surface receptor linked signal transduction	1.39E-67

GO:0008037	cell recognition	1.57E-22

GO:0048610	reproductive cellular process	2.39E-14

GO:0015031	protein transport	1.69E-02

GO:0007264	small GTPase mediated signal transduction	1.34E-25

GO:0007242	intracellular signaling cascade	5.98E-11

GO:0009738	abscisic acid mediated signaling	9.12E-07

GO:0009755	hormone-mediated signaling	2.93E-07

GO:0044070	regulation of ion transport	1.62E-04

GO:0032412	regulation of ion transmembrane transporter activity	1.74E-03

GO:0009788	negative regulation of abscisic acid mediated signaling	4.72E-03

GO:0045454	cell redox homeostasis	5.59E-04

GO:0006470	protein amino acid dephosphorylation	3.46E-18

GO:0010119	regulation of stomatal movement	5.42E-06

GO:0010304	PSII associated light-harvesting complex II catabolic process	3.93E-04

GO:0007243	protein kinase cascade	6.90E-04

GO:0000165	MAPKKK cascade	8.64E-04

GO:0046686	response to cadmium ion	3.22E-09

GO:0010038	response to metal ion	2.54E-06

GO:0009651	response to salt stress	3.76E-09

GO:0009628	response to abiotic stimulus	6.03E-04

GO:0009407	toxin catabolic process	5.16E-11

GO:0045454	cell redox homeostasis	2.37E-06

GO:0016998	cell wall macromolecule catabolic process	1.62E-05

GO:0006469	negative regulation of protein kinase activity	1.37E-03

GO:0009742	brassinosteroid mediated signaling	7.15E-04

GO:0009736	cytokinin mediated signaling	3.71E-07

**Table 3:** **Enriched GO biological process terms involved in predicted *Pseudomonas syringae *proteins**.

GO id	GO term	*p***-value**
GO:0006468	protein amino acid phosphorylation	1.70E-19

GO:0018202	peptidyl-histidine modification	4.51-17

GO:0006796	phosphate metabolic process	2.80E-15

GO:0000160	two-component signal transduction system	3.62E-16

GO:0006355	regulation of transcription, DNA-dependent	2.58E-04

GO:0051252	regulation of RNA metabolic process	2.66E-04

GO:0006935	chemotaxis	1.25E-03

GO:0007626	locomotory behavior	1.25E-03

GO:0018202	peptidyl-histidine modification	2.07E-03
GO:0009405	pathogenesis	7.11E-04

GO:0006026	aminoglycan catabolic process	1.54E-03

GO:0016998	cell wall macromolecule catabolic process	2.67E-03

GO:0052034	negative regulation by symbiont of pathogen-associated molecular pattern-induced host innate immunity	5.03E-04

GO:0000272	polysaccharide catabolic process	9.92E-03

GO:0006022	aminoglycan metabolic process	4.37E-02

GO:0040029	regulation of gene expression, epigenetic	1.65E-03

GO:0016458	gene silencing	1.32E-02

GO:0075343	negative regulation by symbiont of defense-related host callose deposition	1.65E-03

GO:0006342	chromatin silencing	1.32E-02

### Subcellular localization of* Arabidopsis *proteins targeted by the predicted* Pseudomonas *proteins

Pathogens suppress host immunity by directing a range of secreted proteins or effectors, to the cytoplasm of host cells. Once these effector proteins traversed the host plasma-membrane, are transported to many subcellular locations where they subvert the host immune system to enable pathogen growth and reproduction. The knowledge of cellular compartments of the* Arabidopsis *proteins targeted by the predicted* Pseudomonas *will be helpful in deciphering the mechanism of host-pathogen interactions. If the targeted* Arabidopsis *proteins are located in cellular compartments that are very relevant to the pathogen's infection or very likely to be involved in interactions with the pathogen, then the prediction result supports the host-pathogen predictions.

To have a clear understanding the location of the interactions in host, we extracted the subcellular localization of the predicted* Arabidopsis *proteins from both the domain based and interolog methods using the AtSubP [36] available in TAIR database. To date, AtSubP is the only tool for subcellular location prediction of* Arabidopsis *proteins on a genome-scale with high accuracy for seven locations. The subcellular locations of all predicted* Arabidopsis *proteins are listed in Table 4. We found that 29% host proteins are localized in nucleus, 9% in extracellular, 10% in chloroplast, 16% in cytoplasm, 10% in cell membrane, 1% in Golgi, 5% in mitochondrion and 20% as unknown. It reveals that major of the interactions occur in nucleus, cytoplasm, chloroplast and plasma membrane region. In a recent review by Block and James [33] shows that the effectors of *Pseudomonas syringae *target the plant proteins mostly in plasma membrane, chloroplast and mitochondrion. Citovsky et al. [37] showed that when *Agrobacterium tumefaciens *interact with *A. thaliana*, it hijacks VIP1 protein and use it to shuttle transfer-DNA (T-DNA) into the nucleus for its reproduction. Tao et al. investigated that TIP, an* Arabidopsis *protein, interacts with the coat protein (CP) of Turnip crinkle virus (TCV) in yeast cells in nuclei [38]. Thus, the predicted locations of the interacting* Arabidopsis *proteins by our approach are in close agreement with the earlier findings. Also the localizations for a large number of proteins are still unknown which need a special attention for experimental characterization.

**Table 4:** **Distribution of subcellular localization for predicted interacting proteins in *Arabidopsis thaliana *from both the domain and interolog-based approaches**.

Arabidopsis protein location	# of proteins	Percentage (%)
Nucleus	4101	29

Chloroplast	1356	10

Cytoplasm	2317	16

Golgi apparatus	192	1

Mitochondrion	770	5

Extracellular	1199	9

Cell membrane	1321	10

Unknown	2787	20

^* ^Predictions were generated using Arabidopsis-specific subcellular localization predictor, AtSubP (*Plant Physiology *2010, 154: 36-54).

## Conclusion

In this study, we have demonstrated that the sequence and domain similarity to known interactions are valuable information in predicting the host-pathogen interactions. We identified ~11000 PPIs between *Arabidopsis thaliana *and *Pseudomonas syringae *pv. *tomato *DC3000 based on the domain-based and interolog approaches. The functional annotations of both* Arabidopsis *and* Pseudomonas *proteins involved in the predicted PPI are analyzed and it shows the relevance of the proteins for host defense and pathogen infections. The present work may provide some useful information and resource to the plant community to understand the molecular mechanism of the plant immunity system against pathogen virulence. The quality of the predicted interactome could further be improved by combining these methods with other computational approaches and biological data sources. The reliability of the predicted interactions can be further assessed through experimental validations.

## List of abbreviations used

*Pst DC3000, Pseudomonas syringae *pathovar *tomato *DC3000; DIP, Database of Interacting Proteins; HPIDB, Host Pathogen Interaction Database; PPI, protein-protein interaction; GO, Gene Ontology; TTSS, type III protein secretion system; 3DID, three-dimensional interacting domains; iPfam, Protein family interactions; DAVID, Database for Annotation, Visualization and Integrated Discovery.

## Competing interests

The authors declare that they have no competing financial interests.

## Authors' contributions

SSS collected the host and pathogen proteome datasets, developed algorithms and models, performed the calculations, figures and tables, and wrote the draft manuscript. TW helped in data analysis and setting up the pipelines on High-Performance Computing Center. RK conceived the study, participated in its design and coordination, and edited the final manuscript. All authors read and approved the final manuscript.

## Supplementary Material

Additional file 1 Predicted protein-protein interaction pairs for Arabidopsis-*Pseudomonas syringae *based on the domain model. Click here for file

Additional file 2 Predicted protein-protein interaction pairs for Arabidopsis-*Pseudomonas syringae *based on the interolog model. Click here for file

Additional file 3 Consensus protein-protein interaction pairs predicted based on both the domain and interolog models in Arabidopsis-*Pseudomonas syringae*. Click here for file

## Declaration

Funding for the publication of this article has come from OCAST funds account AB-5-42820, OSU.

This article has been published as part of *BMC Bioinformatics *Volume 15 Supplement 11, 2014: Proceedings of the 11th Annual MCBIOS Conference. The full contents of the supplement are available online at http://www.biomedcentral.com/bmcbioinformatics/supplements/15/S11.
